# L-Asparaginase delivered by *Salmonella typhimurium* suppresses solid tumors

**DOI:** 10.1038/mto.2015.7

**Published:** 2015-06-10

**Authors:** Kwangsoo Kim, Jae Ho Jeong, Daejin Lim, Yeongjin Hong, Hyung-Ju Lim, Geun-Joong Kim, So-ra Shin, Je-Jung Lee, Misun Yun, Robert A Harris, Jung-Joon Min, Hyon E Choy

**Affiliations:** 1 Department of Microbiology, Chonnam National University Medical School, Gwangju, Republic of Korea; 2 Department of Molecular Medicine, Chonnam National University Graduate School, Gwangju, Republic of Korea; 3 Department of Biological Sciences, College of Natural Sciences, Chonnam National University, Gwangju, Korea; 4 Department of Hematology-Oncology, Chonnam National University Hwasun Hospital, Jeollanamdo, Republic of Korea; 5 Department of Nuclear Medicine, Chonnam National University Hwasun Hospital, Jeollanamdo, Republic of Korea; 6 Roudebush VA Medical Center and the Department of Biochemistry and Molecular Biology, Indiana University School of Medicine, Indianapolis, Indiana, USA

## Abstract

Bacteria can be engineered to deliver anticancer proteins to tumors via a controlled expression system that maximizes the concentration of the therapeutic agent in the tumor. L-asparaginase (L-ASNase), which primarily converts asparagine to aspartate, is an anticancer protein used to treat acute lymphoblastic leukemia. In this study, *Salmonellae* were engineered to express L-ASNase selectively within tumor tissues using the inducible *araBAD* promoter system of *Escherichia coli*. Antitumor efficacy of the engineered bacteria was demonstrated *in vivo* in solid malignancies. This result demonstrates the merit of bacteria as cancer drug delivery vehicles to administer cancer-starving proteins such as L-ASNase to be effective selectively within the microenvironment of cancer tissue.

## Introduction

Bacterial cancer therapy relies on bacteria that can target solid tumors and proliferate therein (reviewed in refs. 1–3). Although the mechanism underlying this phenomenon has yet to be understood, an immune-privileged environment in tumor tissue should offer a sanctuary for intratumoral bacteria, which can proliferate up to 10^9^ colony forming unit/g tissue.^1,4^ Such bacterial proliferation, especially that of *Salmonella* spp. or *Clostridium* spp., results in tumor regression.^5–7^ Bacteria equipped with anticancer cargo proteins are often more effective tumor suppressors than bacterial monotherapy.^8–10^ Such anticancer proteins include cytotoxic agents, cytokines that stimulate immune cells to kill cancer cells, and tumor antigens that sensitize the immune system against cancer cells.^1,11^ Since most, if not all, of the anticancer proteins are more or less toxic to normal cells, they should be expressed exclusively in intratumoral bacteria.^12–15^

Asparaginase (L-ASNase) of *Escherichia coli* origin is a universal component of therapy for acute lymphoblastic leukemia.^16^ L-ASNase catalyzes the deamination of asparagine to aspartate and to a lesser extent the deamination of glutamine to glutamate.^17^ Both activities may be needed for therapeutic effectiveness against malignancies.^17,18^ Asparagine depletion leads to an adaptive response in which uncharged tRNA activates the serine/threonine kinase GCN2 (ref. 19). GCN2 phosphorylates the translation initiation factor eIF2á, which acts as a dominant inhibitor of the guanine nucleotide exchange factor eIF2B, which prevents eIF2 recycling during protein synthesis, resulting in inhibition of global protein synthesis.^20^ Unless asparagine can be resynthesized rapidly enough to keep its tRNA charged, cells undergo apoptotic cell death for a downregulation of the overall rate of protein synthesis.^21^

The glutaminase activity of L-ASNase also promotes apoptosis. A high intracellular glutamine concentration promotes the uptake of leucine which stimulates protein synthesis by activating mTORC1.^22^ The reduction of mTORC1 activity when glutamine is low suppresses protein synthesis and augments the effects of asparagine deficiency on apoptosis. Furthermore, mitochondrial catabolism of glutamine can rescue tumor cells from asparagine deficiency by providing the four carbons and two nitrogens required for asparagine synthesis.^23^ What’s more, glutamine is required for the resynthesis of asparagine from aspartate by asparagine synthetase (ASNS).^24^ ASNS expression is normally low in cells but activation of the GCN2-eIF2á system promotes translation of the transcription factor ATF4 (ref. 25) which induces ASNS expression. Provided sufficient glutamine is present, ASNS can promote asparagine accumulation which suppresses GCN2 and rescues the cells from apoptosis. L-ASNase has been used successfully to treat blood borne acute lymphoblastic leukemia tumors via intravenous (i.v.) administration^26^ because rescue circuits fail in acute lymphoblastic leukemia cells.^27,28^ Prior to this study, it was rarely possible to treat solid tumors with L-ASNase,^29^ in part because systemic treatment with the high concentrations of L-ASNase needed to affect the asparagine concentration in the tumor is frequently accompanied by serious side-effects including anaphylactic shock, coagulopathies as well as liver and pancreatic toxicity.^30^ Furthermore, it has been thought that upregulation of ASNS would rescue the tumor from apoptosis.^31^ However, in this study, *Salmonellae* was engineered to express large amounts of L-ASNase (EC2) of *E. coli* origin selectively within solid tumors using a remote gene control system derived from *araP_BAD_* inducible by systemic administration lf soluble activator, L-arabinose.^8^ This is the first demonstration of antitumor efficacy of targeted L-ASNasein solid tumor models.

## Results

### Cytotoxicity associated with L-ASNase expressed from *Salmonellae in vitro*

**Figure 1:**
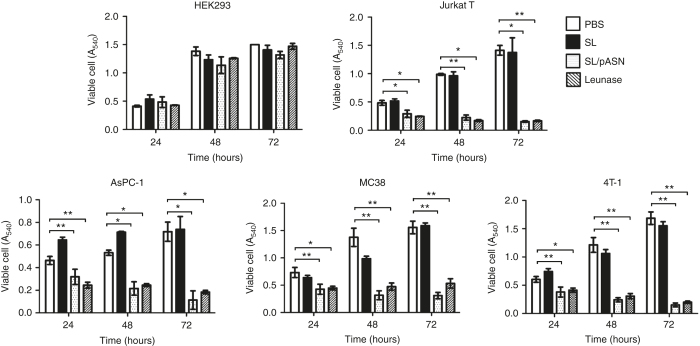
Cell death induced by the L-ASNase present in the bacterial culture medium. *Salmonellae* carrying pASN (SL/pASN) or parental vector *GlmS*^*+*^*p*^32^ were grown in the presence of L-arabinose. The spent medium was collected after centrifugation and filtration, and concentrated to 50 mg protein/ml. Cytotoxicity (200 mg) was tested using cultured animal cells. The assay included 5 units of Leunase as control. Cell death was measured after 24, 48, and 72 hours by MTT assay.^41^ Actual viable cells (A_540_ values) ± SD were plotted as function of time. Asterisks (*) indicate significant differences compared to phosphate-buffered saline (*P = 0.0097, ** P = 0.0119).

In an attempt to treat solid malignancies with L-ASNase, we constructed a plasmid, pASN, carrying the *asnB* gene of *E. coli B* (BL21) under the control of the *araBAD* promoter*(P*_*BAD*_) of the *E. coli* arabinose operon, which is inducible by L-arabinose, by cloning the 1,047 bp PCR-amplified *asnB* open reading frame into *GlmS*^*+*^*p* (Supplementary Figure S1a).^32^ The *GlmS*^*+*^*p* is a balanced lethal host vector system^32^ that relies on the phenotype of the GlmS^-^ mutant. This mutant undergoes lysis when grown in the absence of N-acetyl-D-glucosamine (GlcNac) unless complemented by a plasmid carrying the *glmS* gene.^32^ We used a highly attenuated *S. typhimurium* strain (14028s) defective in ppGpp synthesis^33^and carrying a mutation in the *glmS* gene (ΔppGpp *S. typhimurium*), and transformed it with pASN. The *Salmonellae* were grown in Luria Broth supplemented with L-arabinose (0.2%; ref. 34). The bacterial culture was harvested, the supernatant medium was separated from the cells by centrifugation and filtration, and each fraction was analyzed for L-ASNase by western blotting using a specific antibody (Supplementary Figure S1b). L-ASNase was induced by the L-arabinose supplementation. Moreover, a significant fraction of L-ASNase was secreted, and thus found in the supernatant, as in *E. coli*.^35^ Subsequently, proteins in the culture supernatant were tested for L-ASNase activity by measuring cytotoxicity in three cultured cancer cell lines: mouse MC38 colon cancer cells, mouse 4T-1 breast cancer cells, and human AsPC-1 pancreatic cancer cells (Figure 1). The Jurkat T-cell leukemia line and the immortalized noncancerous HEK293 kidney cell line, sensitive and insensitive, respectively, to L-ASNase, were included as positive and negative controls, respectively. The cells were exposed for 24, 48, and 72 hours to the concentrated culture supernatants (0.2 mg/ml) of *Salmonella* carrying parental plasmid (glmS^+^*p*) or pASN, and cell death was measured by 3-(4,5-dimethylthiazol-2-yl)-2,5-diphenyl-tetrazolium bromide (MTT) assay. All cell lines except HEK293 showed varying degrees of sensitivity (60–80%) to L-ASNase-containing bacterial culture supernatant and to L-ASNase used clinically (Leunase, 5 units/ml, Kyowa Hakko Kirin, Japan). By contrast, only marginal cytotoxicity was observed upon exposure to the culture supernatant of *Salmonella* carrying *glmS*^*+*^*p*, confirming the L-ASNase specificity of the cytotoxicity observed upon exposure to the supernatants of *Salmonella* carrying pASN. It was also noted that the suppression of cell growth by the L-ASNase-containing bacterial supernatant was immediate irrespective of growth rate of the tumors, among those tested (Supplementary Figure S2).

Jurkat T cells treated with L-ASNase have been reported to undergo apoptosis.^36^ In order to assess the cell death mechanism of those tested cancer cell lines, we measured Annexin V, which is associated with apoptotic cell death. Total protein content was extracted from the cells exposed for 36 hours to the L-ASNase-containing bacterial supernatant, and analyzed for Annexin V expression by western blotting. We observed a marked increase in the level of Annexin V in all cancer cells tested (Supplementary Figure S3a). This result was verified by immunofluorescence staining (Supplementary Figure S3b). L-ASNase-containing bacterial supernatant caused a considerable accumulation of Annexin V on the cell surface compared to phosphate-buffered saline (PBS). We concluded that the L-ASNase expressed and secreted by *Salmonellae* induces apoptotic cell death in cancer cells *in vitro*.

### Antitumor efficacy of Salmonella-derived L-ASNase in mouse models

**Figure 2:**
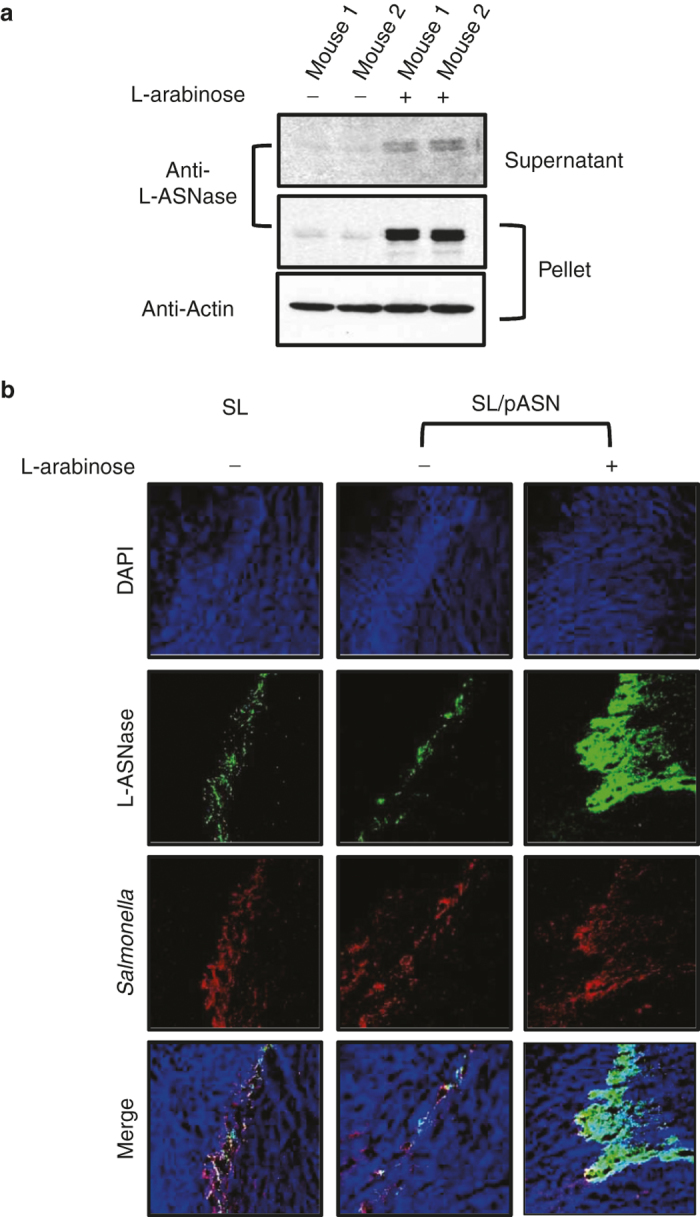
Expression and release of L-ASNase from intratumoral *Salmonellae* carrying pASN. Mice bearing MC38 tumors were injected with *Salmonellae* carrying pASN(SL/pASN). L-ASNase was induced by i.p. injection of L-arabinose (60 mg), and the tumor tissues were excised 8 hours after the induction, homogenized, and centrifuged (5,000 × g, 5 minutes) to obtain the “supernatant” devoid of intact bacteria and the “pellet” containing macromolecules. 100 µg of pellet protein and supernatant protein were run on a 10% SDS PAGE gel and probed with L-ASNase-specific antibody (**a**). The same tissue samples were analyzed by confocal microcopy after immunofluorescence staining of L-ASNase (**b**). *Salmonellae* were stained in red and L-ASNase in green using specific antibodies.

First, we measured the expression of L-ASNase under the control of *P*_*BAD*_ in *Salmonella* targeted to MC38 tumors ectopically grafted in mice. 1 × 10^7^ ΔppGpp *S. typhimurium* transformed with pASN was i.v. injected into the tumor-bearing mice and the L-ASNase was induced by intraperitoneal (i.p.) injection of L-arabinose 4 days postinoculum (dpi). We have not noted any sign of serious local or systemic inflammatory reaction following the induction of cytotoxic antitumor protein in intratumoral *Salmonella* at 3 dpi.^9^ Eight hours after the administration of L-arabinose, tumors (*n* = 2) were harvested, extracted, and the extracts separated by centrifugation into a supernatant containing subcellular molecules and a pellet containing macromolecules including intact bacteria that were analyzed for the expression and secretion of L-ASNase by western blotting (Figure 2a L-ASNase-specific bands(32 kDa) were detected in the animals administered with L-arabinose. Moreover, a considerable amount of L-ASNase was detected in the supernatant, approximately a quarter of the amount found in the pellet. This was further verified by immunohistochemical staining of the tumor tissue samples with L-ASNase-specific antibody (Figure 2b). Significantly elevated levels of L-ASNase were detected in mice treated with *Salmonellae* carrying pASN only after L-arabinose administration. It should be noted that L-ASNase-specific antibody also stained endogenous *Salmonellae* L-ASNase which shares 93% amino acid identity with *E. coli* L-ASNase (*Salmonella* alone), although the signal was weaker.

**Figure 3:**
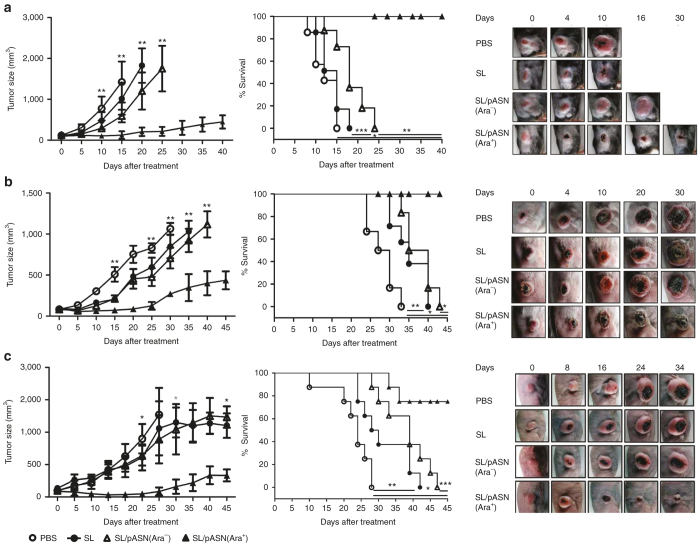
Antitumor effects of *Salmonellae* carrying L-ASNase *in vivo*. The antitumor effect of *Salmonellae* carrying pASN (1 × 10^7^ colony forming unit) was measured using (**a**) MC38 tumors in C57BL/6 mice, (**b**) 4T-1 tumors in BALB/c mice, and (**c**) AsPC-1 tumors in BALB/c athymic nu^-^/nu^-^ mice. The tumor-bearing mice were treated with phosphate-buffered saline (*n* = 6), *Salmonellae* carrying empty vector, *GlmS*^*+*^*p*^32^ (*n* = 6), or *Salmonellae* carrying pASN(SL/pASN) (*n* = 6 each group. L-arabinose (60 mg) was administered i.p. every day starting 4 days after the treatment with engineered *S. typhimurium* (SL/pASN (Ara^+^)). Representative gross morphological changes are shown on the right. Changes in tumor size after the bacterial treatment are shown on the left. Error bars correspond to upper or lower 95% CIs. Kaplan-Meier survival curves of mice bearing MC38 (*P < 0.001, **P = 0.001, ***P = 0.005), 4T-1 (*P < 0.001, **P = 0.0019) or AsPC-1 tumors (*P < 0.001, **P = 0.001, ***P = 0.003) are shown in the middle. Data represent mean ± SD, and asterisks (*) indicate significant differences compared to phosphate-buffered saline controls (*P < 0.05).

Subsequently, we assessed the antitumor efficacy of the *Salmonellae* transformed with pASN in mice bearing tumors: (i) MC38 tumors in C57BL/6 mice, (ii) 4T-1 tumors in BALB/c mice, and (iii) AsPC-1 tumors in BALB/c athymic nu^−^/nu^−^mice (Figure 3). *Salmonellae* carrying either pASN or GlmS^+^*p* were injected through the tail vain when the tumor reached ~120 mm^3^ (ref. 32). L-ASNase was induced by intraperitoneal injection of L-arabinose (60 mg/day/mouse) from 3 dpi, and changes in tumor size were recorded. The results showed the greatest antitumor efficacy with *Salmonellae* carrying L-ASNase induced by L-arabinose administration. We observed an antitumor effect with *Salmonella* monotherapy, which was comparable to 4T-1 or smaller than that observed with *Salmonella* carrying pASN without L-arabinose administration (MC38), but measurably greater than PBS. At the end of the experiment, the mean size of MC38 tumors (at 33 days), 4T-1 tumors (at 35 days), and AsPC-1 tumors (at 45 days) treated with *Salmonellae* expressing L-ASNase was 463.7, 367.4, and 331 mm^3^, respectively, while tumors treated with bacteria carrying pASN without induction, that of bacteria carrying parental plasmid, and that of PBS all reached >1,000 mm^3^. Consequently, survival was substantially prolonged in the animals that received bacteria expressing L-ASNase compared to those in other groups (*P* < 0.001) (middle panels). Taken together, we concluded that the L-ASNase expressed and released from *Salmonellae* was effective at suppressing tumor growth in the mouse models used.

### Correlation between antitumor efficacy and L-ASNase activity

**Figure 4:**
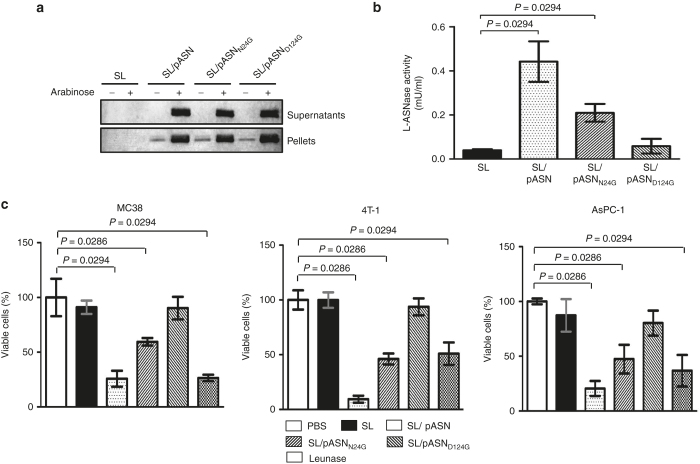
Expression and cytotoxic activity of mutant L-ASNases. (**a**) Expression of two amino acid substitution mutants (see text) and wild-type L-ASNases in the media supernatant and bacterial pellet upon induction with L-arabinose. See legend of Supplementary Figure S1 for details. (**b**) The spent media containing the mutant L-ASNases were assayed for enzyme activity. (**c**) Cytotoxicity associated with these mutant L-ASNases was measured *in vitro* using cultured MC38 (left), 4T-1 (middle), and AsPC-1 (right) by MTT assay. See legend of Figure1 for details. Data represent mean ± SD.

**Figure 5:**
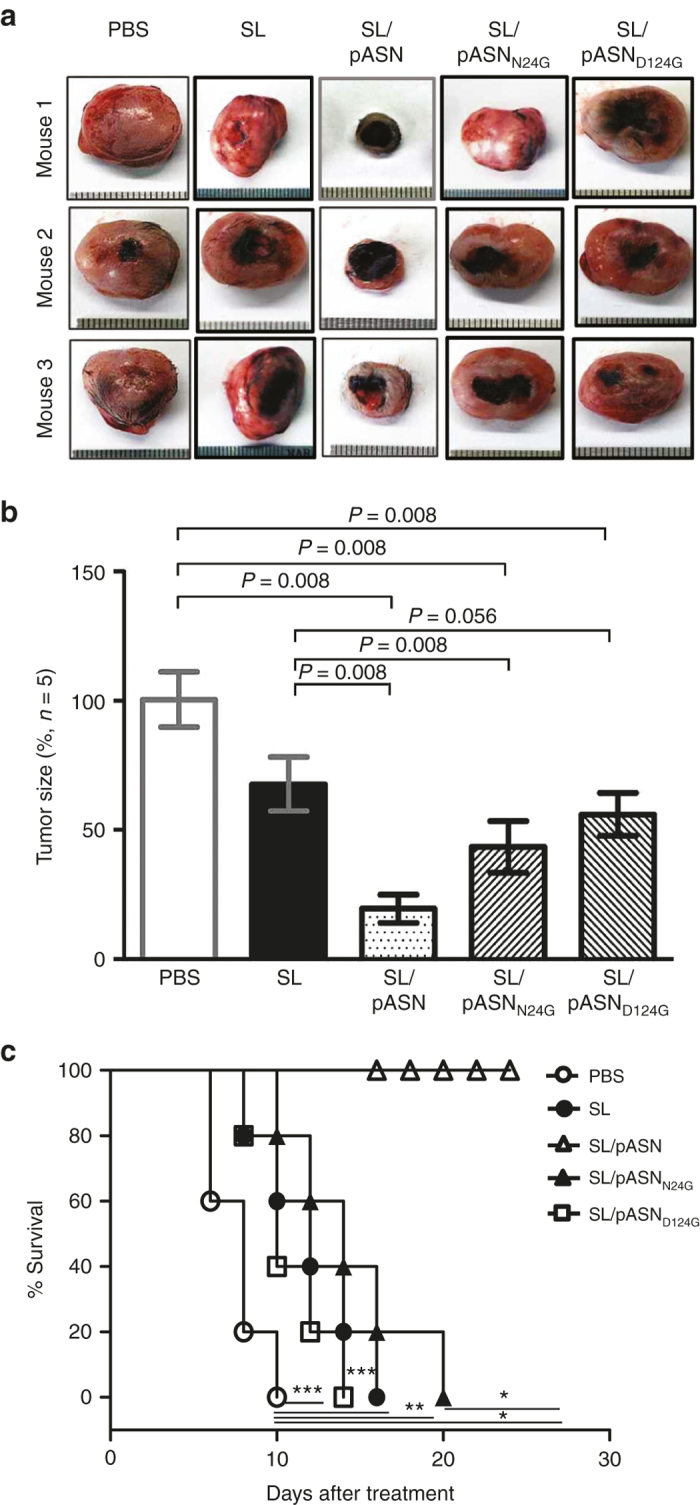
Antitumor effect of the mutant L-ASNases expressed by intratumoral *Salmonellae*. *Salmonellae* (1 × 10^7^ colony forming unit) carrying pASN, pASN_N24G_, or pASN_D124G_, were injected intravenously into C57BL/6 mice carrying MC38 tumors (*n* = 5 each group). (**a**) The change in gross morphology of the tumor tissue after bacterial treatment. (**b**) The changes in tumor size after bacterial treatment and (**c**) Kaplan–Meier survival curves of the tumor-bearing mice. Data represent mean ± SD, and asterisks (*) indicate significant differences compared to phosphate-buffered saline (*P < 0.05).

Since L-ASNase was delivered by *Salmonellae* in this study, it was not possible to determine dose-dependent effect using the conventional method. To this end, we employed mutant L-ASNases defective in enzyme activity. We chose two amino acid substitution mutants carrying Gly in place of Asn^24^ or Asp^124^, N24G and D124G substitution mutants, respectively, reported to have 45 and 3% activity compared to the wild-type protein, respectively.^37^ pASN carrying the above mutations in the *asnB* gene was constructed and named pASN_N24G_ and pASN_D124G_. ΔppGpp *Salmonellae* transformed with these plasmids were grown in LB in the presence or absence of L-arabinose as described previously (Supplementary Figure S1). The bacterial culture was divided into bacterial pellet and medium, and assessed for the presence of ASNase by western blot (Figure 4a). The mutant L-ASNases, as well as the wild-type enzyme, were induced with L-arabinose and secreted into the medium. Subsequently, the bacterial supernatants containing the mutant L-ASNases were assayed for enzyme activity (Figure 4b). Mutant L-ASNases with N24G or D124G substitution showed ~50% and <10% activity, respectively, compared to the wild-type control, as reported previously.^37^ Cytotoxicity was assessed *in vitro* by MTT assay using cultured cancer cells: MC38, 4T-1, and AsPC-1 (Figure 4c). Analysis of the relationship between L-ASNase activities and cancer cell viabilities yielded strong Pearson’s correlations of *r* = −0.97567 (*P* < 0.0001) with MC38, *r* = −0.98707 (*P* < 0.0001) with 4T-1, and *r* = −0.97700 (*P* < 0.0001) with AsPC-1 (Supplementary Figure S4). To test the antitumor effect of mutant L-ASNases *in vivo*, MC38-bearing BL/6 mice were prepared as described previously (Figure 2) and injected through i.v. route with ΔppGpp *Salmonellae* transformed with either pASN_N24G_ or pASN_D124G_. For induction, mice were injected i.p. with L-arabinose at 3 dpi. The gross morphology of tumors extracted at 3 dpi are shown in Figure 5a, and quantification of tumor size in Figure 5b. Animal survival is shown in Figure 5c. A direct correlation between L-ASNase activity and regression of tumor size/extension of survival was noted *in vivo.*

### Correlation between antitumor effect and local concentration of L-ASNase

**Figure 6:**
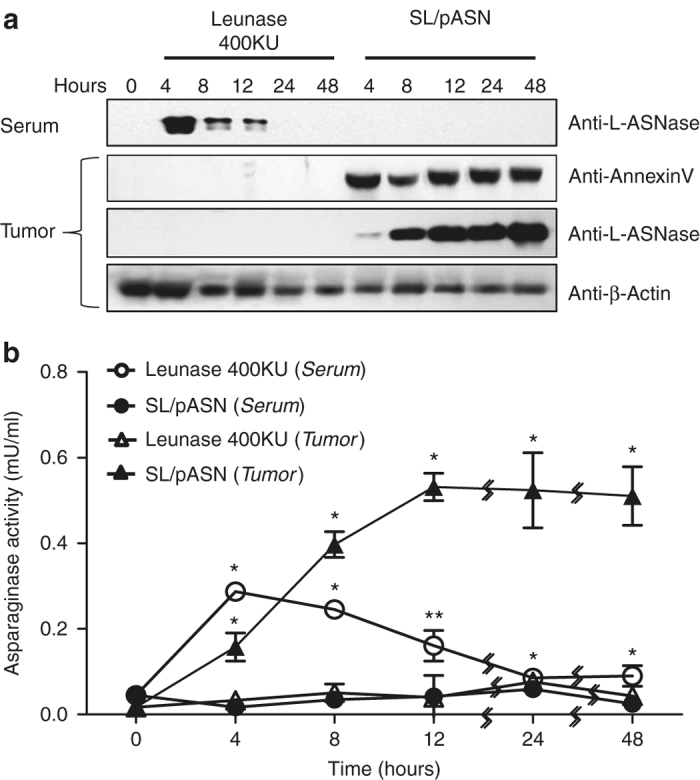
Distribution of L-ASNase in blood and tumor tissue. The C57BL/6 mice bearing MC38 tumors were treated with either 400 KU Leunase or *Salmonellae* carrying pASN (SKS1002/pASN) and analyzed for distribution of L-ASNase. In the latter case, L-ASNase was induced by a single administration of L-arabinose 4 days after bacterial injection (*t* = 0). Whole serum and tumor tissues (100 µg) were collected after the treatment and subjected to quantification of L-ASNase by western blotting (**a**). The same tumor tissues were analyzed for Annexin V and β-actin, the latter as a loading control. (**b**) L-ASNase activity measured using enzyme assay kit in the serum and tumor tissue in the mice treated as described above (*n* = 4 for each group). Data represent mean ± SD.

**Figure 7:**
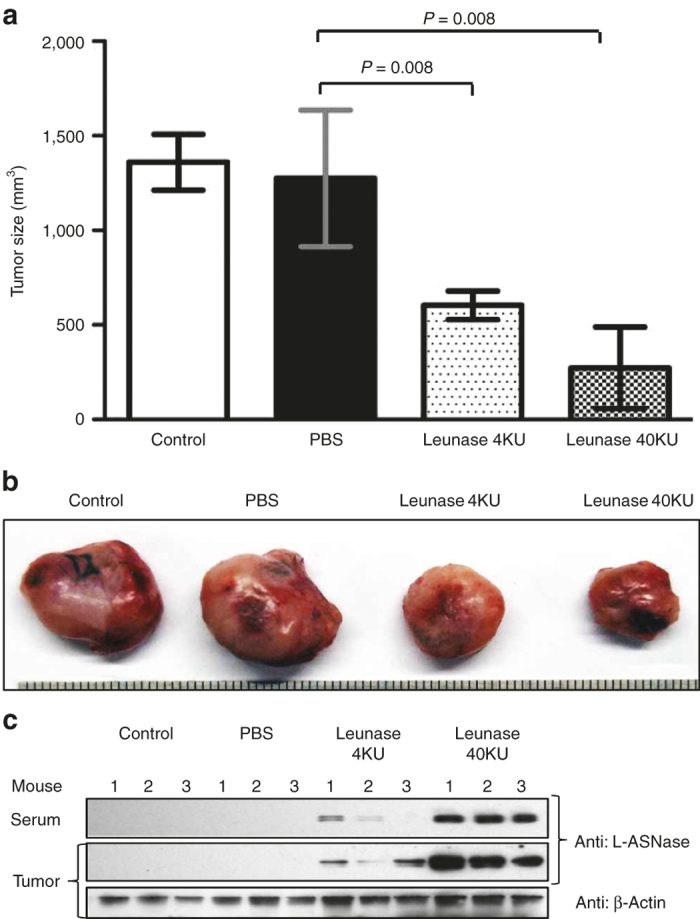
Antitumor effect of Leunase after intratumoral injection. Leunase was directly injected into MC38 tumors in C57BL/6 mice (*n* = 5) at 4 KU and 40 KU daily for 3 days. On day 4, animals were sacrificed and tumor size was estimated as described in the legend of Figure 5 (**a**). (**b**) Gross morphology of the tumor tissues. (**c**) Distribution of L-ASNase in blood and tumor tissue after the Leunase treatment by western blotting. Data represent mean ± SD, and asterisks (*) indicate significant differences compared to phosphate-buffered saline (*P < 0.05).

To understand the distribution of L-ASNase expressed and secreted by tumor-targeted *Salmonellae*, we analyzed serum and tumor tissue in MC38-bearing mice by western blot using L-ASNase-specific antibody (Figure 6a) and by enzymatic activity assay (Figure 6b). Leunase (400 KU) injected intravenously was used as a control. Tissue samples were collected over time after administration of L-arabinose into mice treated with *Salmonellae* or after Leunase administration. In the animals treated with Leunase, a considerable amount of L-ASNase was detected in the serum at the early time point (4 hours) and diminished over time, but none was detected in the tumor tissue. By contrast, in mice treated with *Salmonellae* expressing L-ASNase, the protein was detected only in tumor tissue and increased dramatically as time progressed up to 48 hours. Accordingly, annexin V was induced in the tumor tissue only when the mice received *Salmonellae* expressing L-ASNase. Intravenous administration of Leunase to MC38-bearing mice did not reduce tumor size (Supplementary Figure S5a and a high dose (400KU) shortened animal survival (Supplementary Figure S5b, which should be ascribed to the side effects.^30^ By contrast, a direct intratumoral injection of Leunase resulted in a notable reduction of tumor size (Figure 7a) (for Leunase 40KU versus control group, difference = 336 mm^3^, 95% CI = 220–451, *P* = 0.008; for Leunase 4KU versus control group, difference = 607 mm^3^, 95% CI = 532–681, *P* = 0.008), as it was detectable at the tumor tissues by western blotting (Figure 7). Taken together, we concluded that L-ASNase can be used to treat solid malignancies if delivered by a carrier such as *Salmonellae* to intratumoral sites so that a copious concentration is maintained *in situ*.

## Discussion

Many tumors exhibit deficiencies in the synthesis of one or more amino acids, forcing a reliance on extracellular pools of these amino acids for protein biosynthesis. Therefore, depletion of such amino acid(s) would provide cancer cell-selective therapy with few side effects. L-ASNase is used to mediate the degradation of such amino acids for therapeutic purposes; however, undesirable toxicity hampers systemic injection.^30^ We have also seen a deleterious effect of high-dose L-ASNase injected intravenously (Supplementary Figure S5b). This could be caused by the fact that L-ASNase prevents several forms of glycosylation, including sialylation, in newly synthesized proteins.^38,39^ In addition, *E. coli* L-ASNase possesses glutaminase activity, which results in deprivation of L-glutamine.^39^ Whereas, bacterial tumor therapy circumvents the systemic injection of high-dose L-ASNase, thereby avoiding systemic toxicity while maintaining sufficient levels inside the tumor tissue.

Successful bacterial antitumor therapy, in which intratumoral bacteria are used to express anticancer proteins, requires certain provisions, as follows. First, the antitumor protein should be expressed selectively at the tumor site, as demonstrated in this study. Since bacteria administered intravenously initially localize to reticuloendothelial organs,^8,9^ the protein drugs should be expressed only when the bacteria have cleared reticuloendothelial organs and accumulated exclusively in the targeted tumor tissue; this normally occurs 3 days after administration. We have shown that controlled expression of cytotoxic proteins at 3 dpi does not cause any notable systemic toxicity.^9^ Second, the antitumor protein must be released. Fortunately, a significant fraction of the L-ASNase of *E. coli* origin was secreted from *Salmonellae*.  *E. coli* L-ASNase (EC2) is a periplasmic enzyme that contains a typical secretary signal peptide of 22 residues at its amino terminus. When the enzyme is secreted to the periplasm, the signal peptide is removed to yield a mature protein with an N-terminal leucine residue.^35^ Proteins secreted into the periplasm tend to leak out through the bacterial outer membrane. Lastly, but most importantly, antitumor proteins should act on the cancer cell surface or the tumor microenvironment, rather than on intracellular targets. In the latter case, the barely permeable cell membrane remains a formidable barrier to efficacy. A widely used strategy uses cell-penetrating peptides (CPPs) to improve intracellular uptake. Successful intracellular delivery of antitumor proteins using this strategy requires optimization of CPP for individual anticancer proteins.^12^ Given these considerations, L-ASNase is an anticancer cargo protein that is appropriate for delivery to the tumor microenvironment by tumor-targeting bacteria.

We demonstrated a linear relationship between *in vitro* cytotoxicity and *in vivo* tumor regression using mutant L-ASNases with varying levels of activity (Figures 4 and 5). The linear relationship revealed the dose-dependent effect of L-ASNase *in vivo* and was comparable with that determined using cultured cells *in vitro*. If bacterial therapy with ASNase-expressing *Salmonellae* was to be employed post-surgical elimination of the tumor, prognosis could be assessed based on the *in vitro* sensitivity of the cancer tissue to L-ASNase. In practice, bacterial therapy with *Salmonella* expressing L-ASNase, would be useful to treat cancer patients with multiple organ metastases since bacteria would be capable of targeting tumor of any kind with size as small as 100 mm.^34^

## Materials and Methods

### Plasmid construction

DNA encoding ASNase II (*asnB*) was amplified from the genomic DNA of *E. coli B* strain (BL21)^40^ using two sets of primers: L-ASN1 (5′-GG GAA TTC ATGGAGTTT TTC AAA AAG-3′) and L-ASN2 (5′-GGG TCT AGA TTA GTA CTG ATT GAA GAT CTG CTG-3′). The 1,047 kb DNA fragment of *asnB* was inserted into *GlmS*^*+*^*p*^32^ using *Eco R*I and *Xba *I restriction enzyme sites to generate pASN. The mutant L-ASNases were generated by site-directed mutagenesis (Stratagene, San Diego, CA) using pASN and the following primers: P1, 5′-GACTCCGCAACCAAATCTGGCTACACAGTGGGTAAAG-3′ for N24G; and P2, 5′-ACGTCTATGAGCGCAGGCGGTCCATTCAACCTGT-3′ for D124G. All constructs were verified by automated DNA sequencing.

### Bacterial growth conditions

Bacteria were grown in LB medium (Difco Laboratories) containing 1% NaCl. For solid support medium, 1.5%-bacto agar was included. All media were supplemented with antibiotics as follows: ampicillin at 100 µg/ml, kanamycin at 50 µg/ml, and chloramphenicol at 10 µg/ml.

### Cell culture

MC38 and 4T-1 cell lines were grown in high-glucose Dulbecco’s modified Eagle medium, HEK293 in EME medium, and AsPC1 and Jurkat T in RPMI1640 medium, containing 10% FBS and 1% penicillin-streptomycin.

### Protein (L-ASNase) preparation

The spent bacterial media and bacterial pellets were prepared as follows after centrifugation (5,000 ×g, 5 minutes) and filtration (0.45 µm): the pellets were lysed by sonication in lysis buffer (10 mmol/l lysozyme, 10% sodium dodecyl sulfate (SDS)), and the supernatants were filtered (0.22 µm pore filter) and concentrated using Centricon devices (Amicon^R^ Ultra, 10K pore filter, Millipore). The proteins were precipitated with 10% trichloroacetic acid (1 hour, 4 °C) and dissolved in PBS. Leunase was purchased from Kyowa Hakko Kirin, Japan.

### Western analysis

Mammalian cell lines and tumor tissues were lysed by sonication in RIPA buffer (50 mmol/l Tris-HCl pH 7.5, 150 mmol/l NaCl, 1% NP40, 0.5% sodium deoxycholate, and 0.1% SDS) containing protease inhibitors (protease inhibitor mixture; Roche Applied Science). Tumor tissue were extracted, homogenized with a motor-driven tissue homogenizer (Ika-Werke, Staufen, Germany), and separated to supernatant and pellet by centrifugation (5,000 × g, 5 minutes). The pellets were lysed by sonication as described above. The supernatants were treated with 10% trichloroacetic acid (1 hour, 4 °C) for precipitation of proteins. For western blot analysis, protein samples were boiled for 5 minutes, separated by 10% SDS-PAGE, and transferred to nitrocellulose membrane (Amersham Biosciences). The membrane was blocked with 5% skim milk and probed with a rabbit anti-L-ASNase antibody (1:1,000; Abcam), rabbit anti-Annexin V antibody (1:2,000, Abcam), or mouse anti-β-Actin antibody (1:5,000, Santa Cruz) at 4 °C overnight. The membrane was then incubated with anti-mouse or anti-rabbit IgG linked to horseradish peroxidase (Sigma-Aldrich, UK) for 1 hour and bound proteins were visualized by ECL (Amersham Biosciences).

### Cell viability assay (MTT)

The MTT assay was performed to evaluate cell proliferation. The cells (3 × 10^3^ per well) were seeded into 96-well culture plates in 100 µl medium at 37 °C with 5% CO_2_. After overnight incubation, the supernatant was removed and fresh medium was added. The cells were incubated with concentrated bacterial supernatant (0.2 mg/ml) and the number of live cells was determined using MTT assay kit (Sigma-Aldrich, St. Louis, MO).

### Immunofluorescence and confocal microscopy

The cells were grown on sterilized glass cover slips, fixed with 4% paraformaldehyde, and blocked with 0.1% BSA (bovine serum albumin) in PBS. Subsequently, the samples were stained with rabbit anti-Annexin Va ntibody (1:200, Abcam) in PBS and Alexa 488-conjugated secondary antibody (1:500, Invitrogen). The samples were also stained with Texas Red-X phalloidin (1:500, Invitrogen) and DAPI/antifade (1:200, Invitrogen). Images were captured with a Bio-Rad confocal microscope (Bio-Rad laboratories, Hercules, CA).

### Tissue immunofluorescence staining

Tumor tissues were fixed with 4% paraformaldehyde in PBS overnight at room temperature, and embedded and frozen using optical optimal cutting temperature compound (Tissue-Tek). The tumors were sliced into 6-µm thick sections using a microtome-cryostat. The tissue sections were collected on aminopropyltriethoxysilane-coated slides. The slides were washed using PBS (pH7.4) for complete removal of the optimal cutting temperature compound and incubated with primary antibodies, rabbit anti-L-ASNase antibody (1:100, Abcam), and mouse anti-*Salmonella* antibody (1:100, Abcam), overnight at 4 °C. Alexa 488-conjugated goat anti-rabbit antibody (1:100) and Alexa 568-conjugated goat anti-mouse antibody (1:100) were used as secondary antibodies. The samples were mounted with DAPI/antifade (1:200, Invitrogen).

### Animal experiments

Five- to 6-week-old male mice (20–30 g body weight) were purchased from Samtako Company, Korea. All animal care, experiments, and euthanasia were performed in accordance with the protocols approved by the Chonnam National University Animal Research Committee. Animals were anesthetized with isoflurane (2%) for imaging or a mixture of ketamine (200 mg kg^−1^) and xylasine (10 mg kg^−1^) for surgery. Mice carrying subcutaneous tumors were generated as follows: tumor cells cultured *in vitro* were harvested, suspended in 100 µl PBS, and injected subcutaneously into the right thigh (1 × 10^6^ cells for 4T-1 and MC38, and 1 × 10^7^ cells for AsPC1). Tumor volumes (mm^3^) were estimated using the formula (*L* × *H* × *W*)/2 where *L* is the length, *W* is the width, and *H* is the height of the tumor in millimeters. 1 × 10^7^ ΔppGpp *S. typhimurium* transformed with pASN suspended in PBS was injected through the tail vain when the tumor reached ~120 mm^3^. L-ASNase was induced by intraperitoneal (i.p.) injection of L-arabinose 4 days postinoculum (dpi) (60 mg/day/mouse).

### Injection of bacteria into animals

*S. typhimurium* suspended in 100 µl PBS were injected intravenously into tumor-bearing mice through the lateral tail vein using an l cc insulin syringe.

### Determination of L-ASNase activity

Bacterial supernatants of native and mutants L-ASNase were prepared as concentrated forms for the enzymatic assay. Serum samples were directly used for the enzyme assay. Tumor tissue samples were extracted by the  4 volumes of assay buffer in Asparaginase Activity Assay kit (Abcam, ab107922), and insoluble materials were removed by the centrifugation. Levels of L-ASNase enzymatic activity in the bacterial supernatants (100 µg), serum (10 µl), and tumor tissues (100 µg) were assessed with the Asparatinase Activity assay kit, for a quantitative enzyme assay.

### Correlative analysis

Pearson’s correlation coefficients in Supplementary Figure S5 were computed for the relationship between L-ASNase activities and cell viabilities using GraphPad Prism 5.01, including *P* values for the one-tailed test of significance

### Statistical analysis

Statistical analysis was performed using the SPSS 18.0 statistical package (SPSS, Chicago, IL). A two-tailed Student’s *t-*test was used to assess the statistical significance of tumor growth differences between treatment groups. *P* < 0.05 was considered statistically significant.
