# Supplementary figures and images for: L-Asparaginase delivered by Salmonella typhimurium suppresses solid tumors
Source: Mol Ther Oncolytics. 2015 Jun 10;2:15007–. doi: 10.1038/mto.2015.7 (PMC4845971; doi:10.1038/mto.2015.7)

A

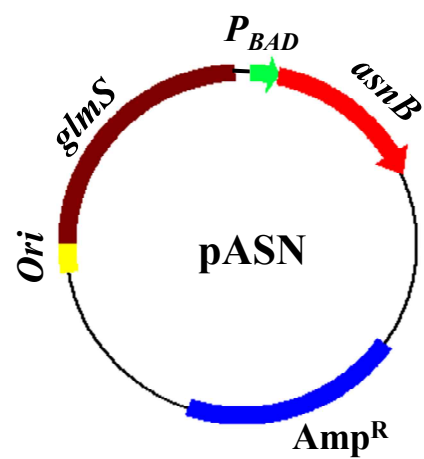

B

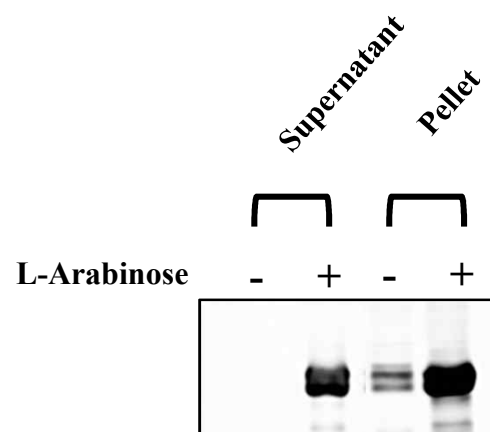

Supplement: Supplementary Figure S1 [file mto20157-s1.pdf]

A

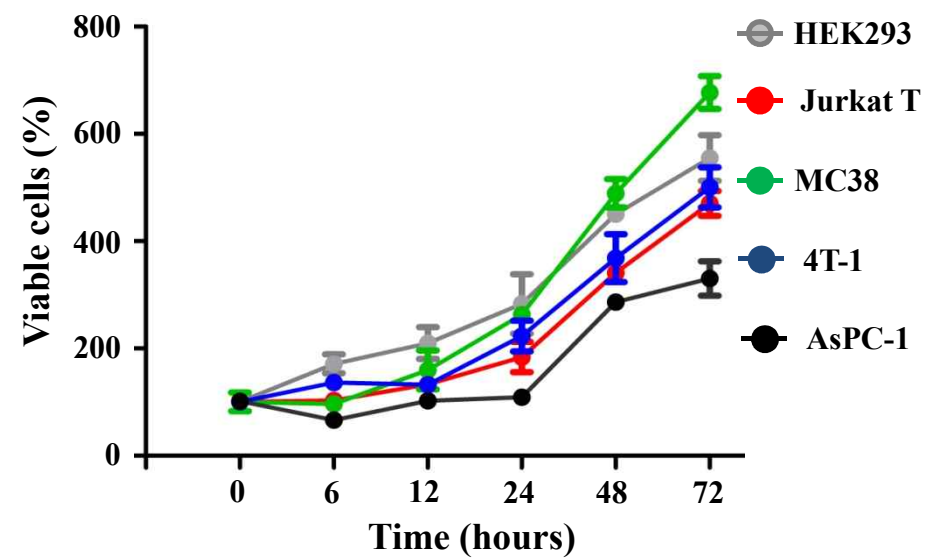

B

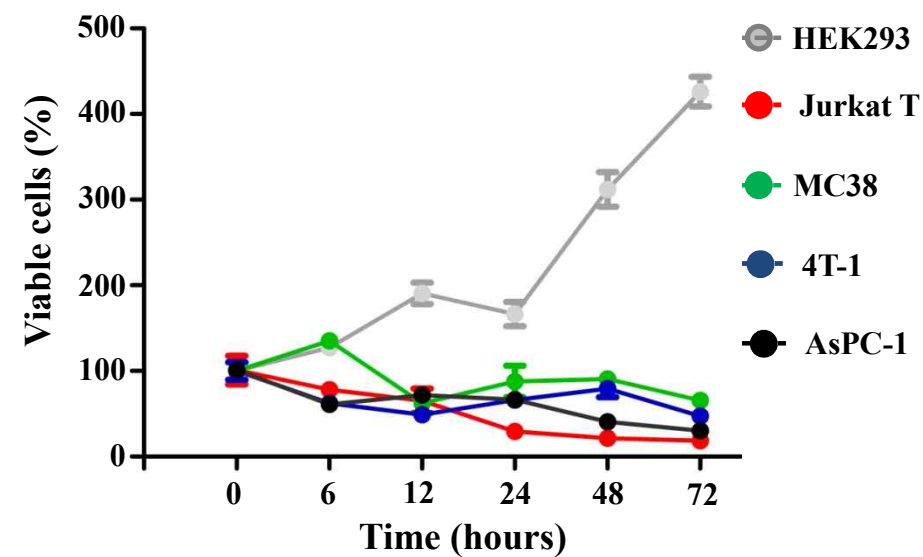

Supplement: Supplementary Figure S2 [file mto20157-s2.pdf]

**A**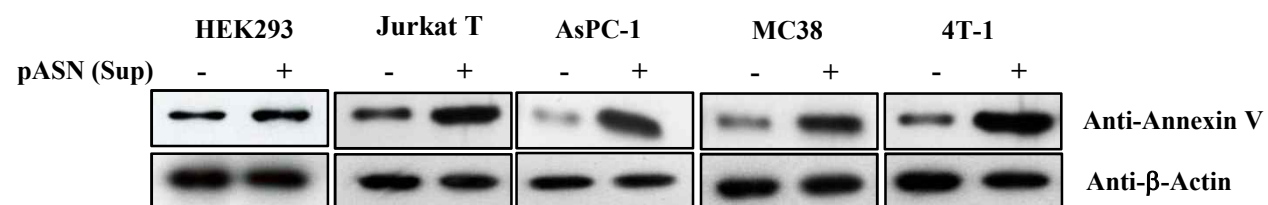**B**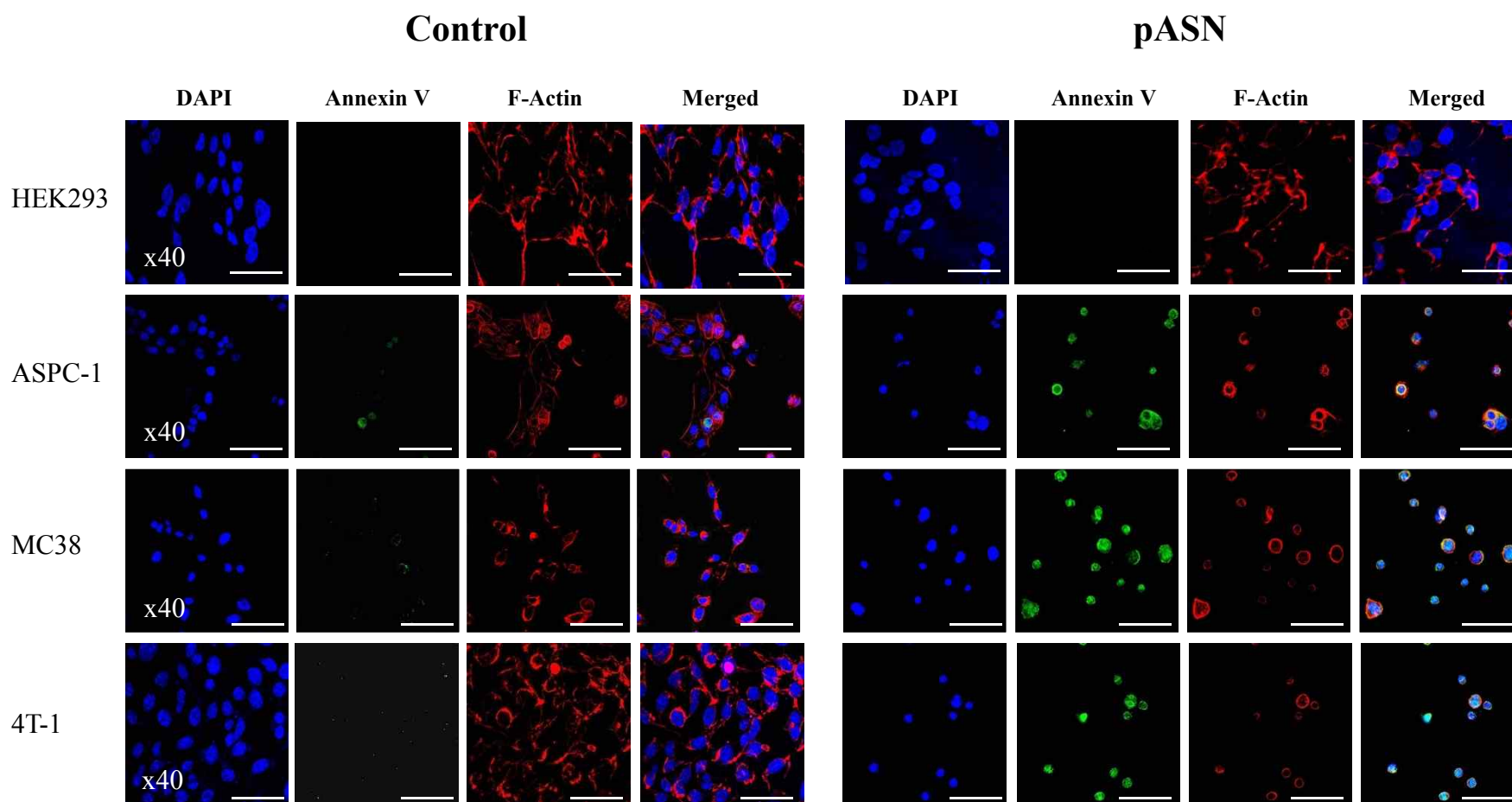

Supplement: Supplementary Figure S3 [file mto20157-s3.pdf]

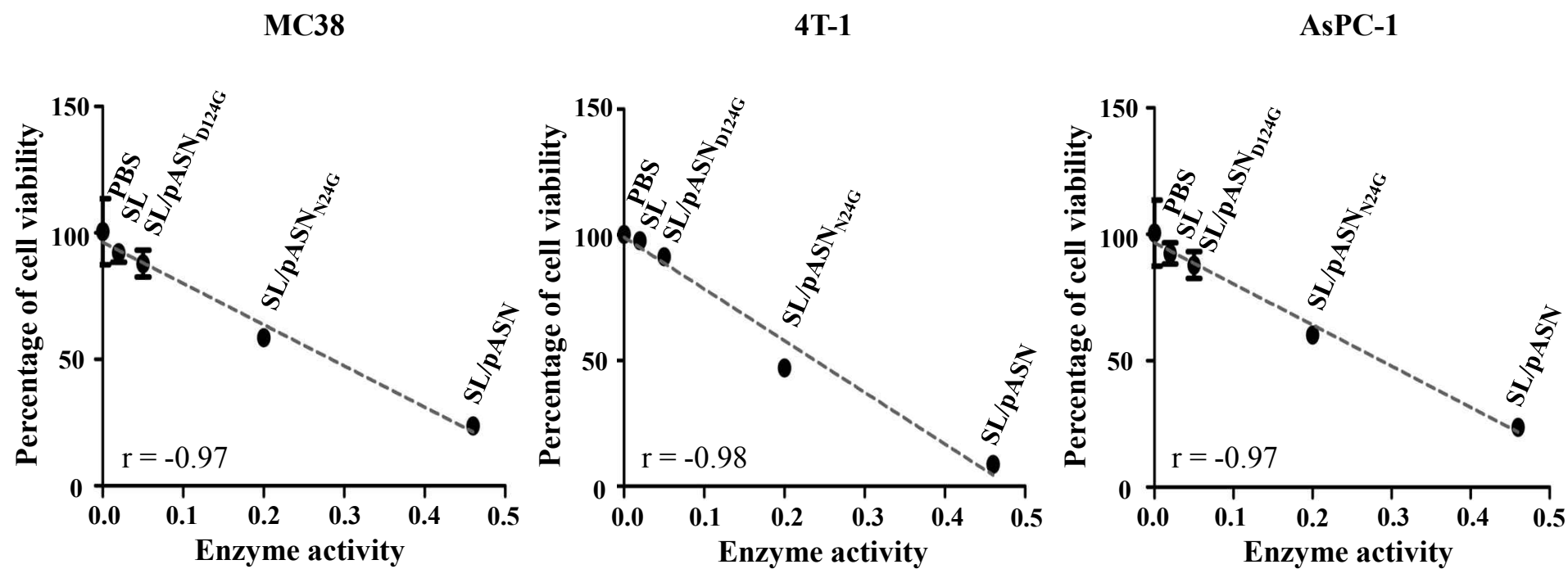

Supplement: Supplementary Figure S4 [file mto20157-s4.pdf]

A

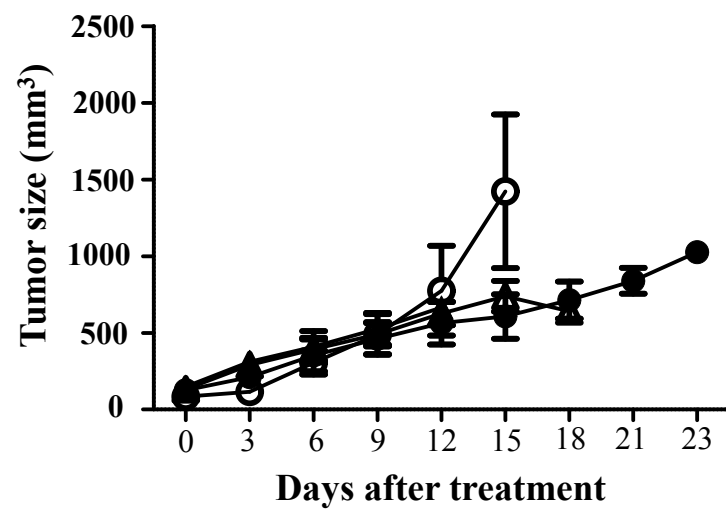

B

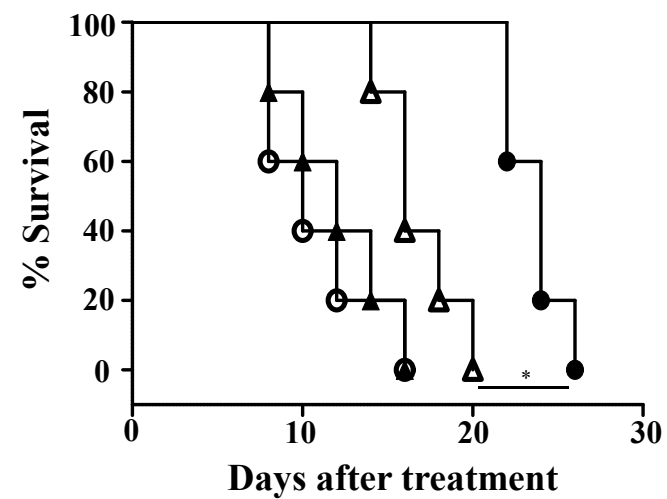

○ PBS    ● Leunase® 4KU    ▲ Leunase® 40KU    ▲ Leunase® 400KU

Supplement: Supplementary Figure S5 [file mto20157-s5.pdf]
